# A case of fat-forming solitary fibrous tumor that is prone to be confused with liposarcoma

**DOI:** 10.1186/s13000-024-01463-8

**Published:** 2024-02-22

**Authors:** Yi-Dan Ma, Zi-Qing Wu, Xin-Rong Liang, Lin Jing Pi, Meng-Zhen Gong, Yao Tang

**Affiliations:** 1 Department of Pathology, Integrated Hospital of Traditional Chinese Medicine, Southern Medical University, Guangzhou, 510315 China; 2 Department of Obstetrics and Gynaecology, Yanshi Maternal and Child Health Hospital, Luoyang, 471900 China

**Keywords:** Fat-forming solitary fibrous tumor, Liposarcoma, Spindle cell lipoma, Retroperitoneal

## Abstract

Fat-forming solitary fibrous tumor is a rare and specific subtype of solitary fibrous tumor. In this case, a mass of 8.3 cm in diameter was found in a 59-year-old male patient’s right retroperitoneum, as revealed by abdominal contrast-enhanced computed tomography (CT) images. The tumor exhibited a well-circumscribed nature and histological features characterized by a combination of hemangiopericytomatous vasculature and mature adipose tissue, comprising around 70% of the total tumor composition. Immunohistochemistry staining revealed diffuse positive expression of STAT6 and CD34 in the tumor cells. Based on these findings, the final diagnosis was determined to be a fat-forming solitary fibrous tumor located in the retroperitoneum. It is important to consider other potential differential diagnoses, including angiomyolipoma, dedifferentiated liposarcoma, spindle cell lipoma, and atypical lipomatous tumor/well-differentiated liposarcoma.

## Introduction

In 1995, Nielsen et al. reported a tumor composed of mature adipocytes and hemangiopericytoma-like areas, which they named lipomatous hemangiopericytoma [1]. In 2000, Guillou et al. noted that lipomatous hemangiopericytoma and solitary fibrous tumor (SFT) share similar clinicopathologic, immunohistochemical, and ultrastructural features, except for the presence of mature adipocytes. They suggested that lipomatous hemangiopericytoma represents a fat-containing variant of SFT [2]. The World Health Organization (WHO) classification classified it as a morphological variant of solitary fibrous tumor (SFT) until 2013 [3].

Fat-forming SFT tends to occur predominantly in the retroperitoneum and deep soft tissues of the lower extremity. To date, 7 cases of retroperitoneal fat-forming SFT have been reported in the previous English literature (summarized in Table 1). When the tumor is located in the retroperitoneum and contains a significant amount of mature adipocytes, it can be easily misdiagnosed as liposarcoma. Liposarcoma, being a malignant tumor, requires extended resection, and postoperative adjuvant treatment. In contrast, fat-forming SFT only requires complete tumor resection without further anti-tumor treatment. Therefore, misdiagnosis can lead to unnecessary overtreatment for patients.

**Table 1:** Summary of clinicopathologic data for reported cases of retroperitoneum fat-forming solitary fibrous tumors

Author/Publication year	Sex/Age (years)	Size(cm)	Treatment	Follow-Up	Diagnosis
this case	M/59	8	SE	NED 10 months	Fat-Forming Solitary Fibrous Tumor
Nielsen et al/1995	M/72	10	SE	NA	Lipomatous Hemangiopericytoma
Folpe et al/1999	M/53	NA	NA	NED 7 years	Lipomatous Hemangiopericytoma
Folpe et al/1999	M/33	18	NA	NA	Lipomatous Hemangiopericytoma
Guillou et al/2000	M/54	7.5	WE	NED 72 months	Deep fibrous histiocytoma
Guillou et al/2000	M/46	19	SE	NED 6 months	Malignant Hemangiopericytoma
Guillou et al/2000	M/51	18	SE	NED 6 months	SFT with fat
Lee et al/2011	F/93	6	SE	NED 7–8 months	Malignant Fat-Forming Solitary Fibrous Tumor

Abbreviations: F, female; M, male; NA, not available; NED, no evidence of disease; SE, surgical excision; WE, wide excision

In this report, we present a case of a 59-year-old male patient with a retroperitoneal fat-forming SFT. We analyze its clinicopathological features with the aim of improving understanding of this tumor and reducing overtreatment due to misdiagnosis.

## Case report

**Fig. 1:**
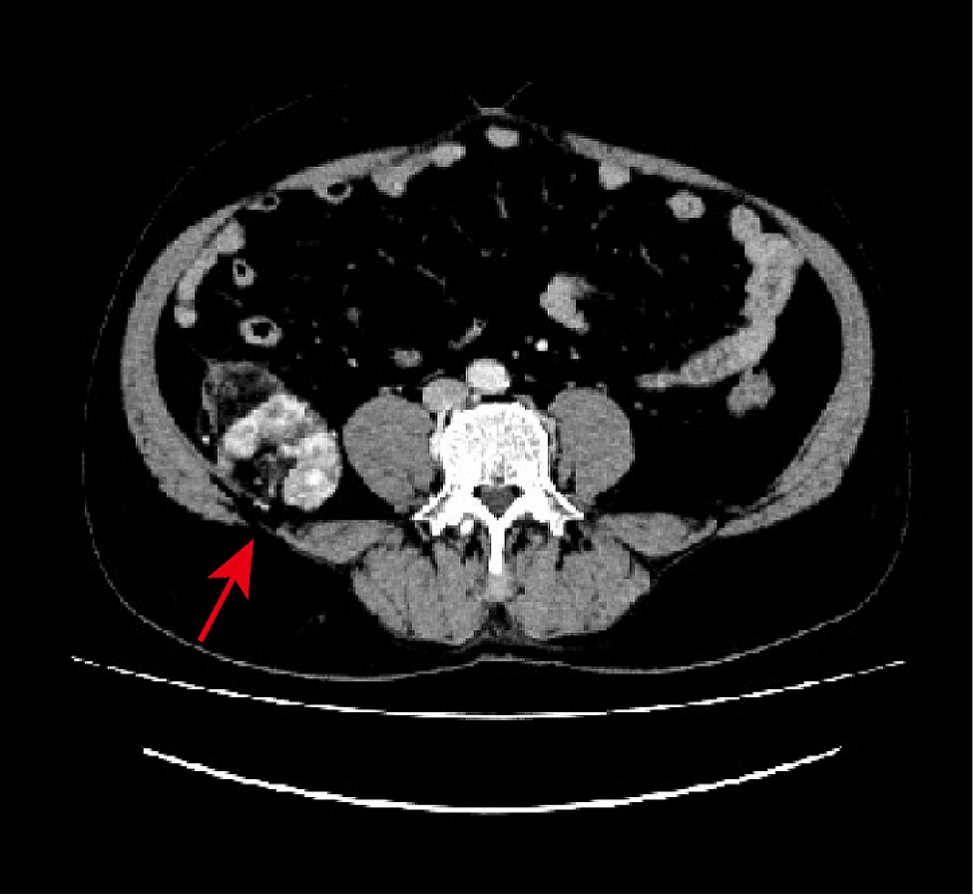
Contrast-enhanced computed tomography (CT) images clearly demonstrate a mass with predominantly fat attenuation adjacent to the right margin of the psoas major muscle in the retroperitoneum (red arrow). The mass exhibits irregular and nodular soft tissue, which displays significant enhancement on contrast-enhanced CT

During physical examination, a mass adjacent to the right margin of the psoas major muscle was detected in a 59-year-old male patient. The patient did not present with any additional symptoms. Contrast-enhanced computed tomography (CT) images showed a mass predominantly composed of fat, measuring approximately 83 × 55 × 81 mm, adjacent to the right margin of the psoas major muscle in the retroperitoneum. Irregular and nodular soft tissue was observed inside the mass, with significant enhancement on CT (Fig. 1). The boundary of the mass was unclear, extending into the abdominal cavity with an indistinct demarcation from the caecum. Based on these imaging findings, the patient initially received a diagnosis of liposarcoma. Subsequently, the mass was surgically excised and sent for histological examination.

**Fig. 2:**
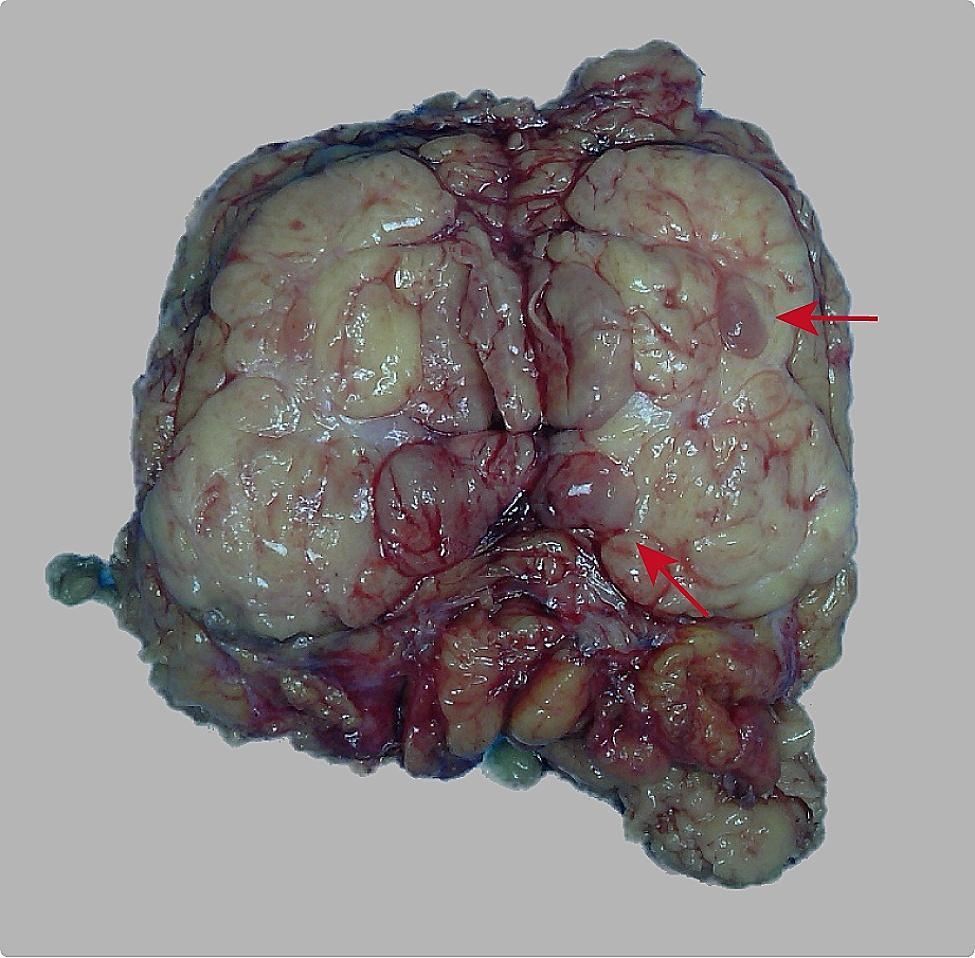
Grossly, a single oval-shaped tumor measuring approximately 80 × 50 × 43 mm was observed. The tumor appeared to be well-encapsulated with a smooth surface. On the tumor section, a vaguely nodular structure (red arrow) with a light red color and tougher texture can be seen, while the remaining areas exhibited a yellow color and soft texture

The cut surface of the specimen revealed an oval-shaped tumor measuring approximately 80 × 50 × 43 mm. The tumor appeared well-encapsulated with a smooth surface and exhibited a vaguely nodular structure with a light red color and tougher texture. In contrast, the remaining areas of the tumor had a yellow color and a soft texture (Fig. 2).

**Fig. 3:**
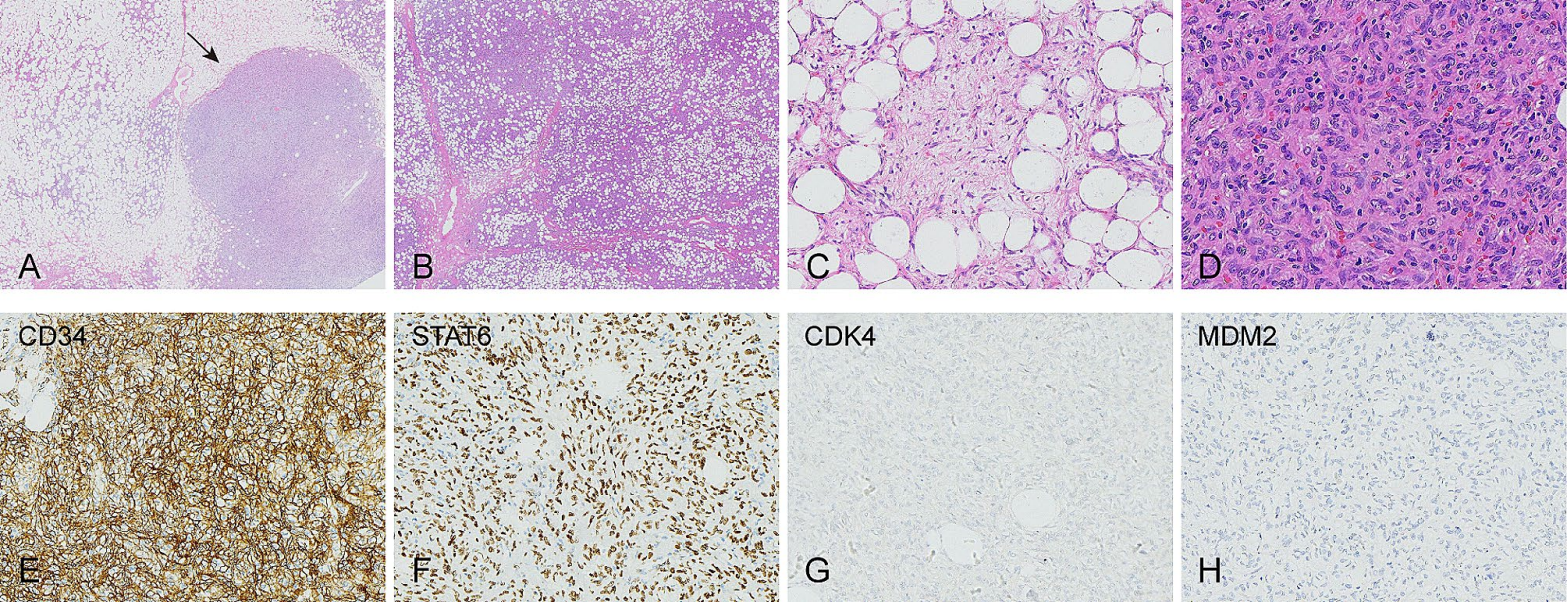
Hematoxylin and eosin (HE) and Immunohistochemical (IHC) staining of the fat-forming solitary fibrous tumor. The tumor consisted of mature adipose tissue and cellular nodules (black arrow) with the classic appearance of SFT. (HE, original magnification (**A**) 20x, (**B**) 20x, (**C**) 200x, (**D**) 400x). Positive immunostaining was observed for (**E**) CD34 and (**F**) STAT6. Negative immunostaining was observed for (**G**) *CDK4* and (**H**) *MDM2*

The microscopic examination revealed a well-circumscribed mass with a fibrous capsule. The mass was composed of cellular nodules with the typical histologic features of SFT, admixed with approximately 70% mature adipose tissue (Fig. 3a). Fibrous septa composed of collagen and large blood vessels were observed in certain areas of the tumor, separating them (Fig. 3b). The characteristic of SFT included ovoid to spindle-shaped tumoral cells arranged in intersecting fascicles or a patternless jumble. These cells exhibited indistinct borders, scant cytoplasm, uniform elongated or fusiform nuclei, and occasional nucleoli (Fig. 3d). Focal myxoid and edematous stromal changes with lower cell density were found (Fig. 3c). In addition, staghorn-shaped blood vessels, with spindle-shaped and oval-shaped cells arranged around them, were evident. No signs of hemorrhage or necrosis were identified in the tumor.

Immunohistochemical staining demonstrated diffuse positivity for STAT6 (Fig. 3f) and CD34 (Fig. 3e) in the spindle cells. The adipose component showed positive staining for S100, while CD31, ERG, Desmin and Actin were positive in the vascular and perivascular cells. CK, *MDM2* (Fig. 3h), *CDK4* (Fig. 3g), Melan A, and HBM45 staining were negative. Fluorescence in situ hybridization (FISH) did not detect amplification of the *MDM2* and *CDK4* genes. Based on the observed histological features and immunophenotype, the accurate diagnosis of fat-forming SFT was established, and other differential diagnoses such as liposarcoma, angiomyolipoma, and spindle cell lipoma were ruled out.

In our case, the lack of necrosis and rare mitotic figures indicated a low-risk classification despite the patient being over 50 years old and tumor size exceeding 5 cm. Although lacking aggressive histologic features, we still recommend close and long-term monitoring based on factors such as the patient’s age, tumor location, and size. It is reassuring to note that 10 months after tumor resection without additional anti-tumor treatment, the patient remains in good health with no signs of recurrence or metastasis.

## Discussion

Solitary fibrous tumor (SFT) is a rare mesenchymal tumor with intermediate behavior, which may occur at any age and in any anatomical location. This includes the pleura, superficial and deep soft tissues, and even within visceral organs and bones [4]. A less common subtype of SFT is the fat-forming solitary fibrous tumor, which typically develops in the retroperitoneum and deep soft tissues of the lower extremities, particularly the thigh [2]. This subtype of SFT typically presents in middle-aged adult males with slow-growing, painless masses [3]. The tumor may be asymptomatic or cause various degrees of local compressive symptoms based on its size and location.

The radiographic features of fat-forming SFT are largely similar. Computed tomography (CT) scans reveal a well-defined, enhanced, hypervascular mass with a significant area of fatty component [5]. These features can contribute to a broad range of differential diagnoses, including adipocytic tumors such as liposarcoma and spindle cell lipoma. In fact, the initial clinicoradiological diagnosis in our case was liposarcoma.

Histologically, SFT presents various morphological patterns, leading Machado et al. to describe them as “the great simulator” of soft tissue tumors [6]. Classical SFT typically exhibits patternless or storiform proliferation, with spindled to ovoid cells featuring indistinct, pale eosinophilic cytoplasm. These cells are found within a collagenous stroma, admixed with branching and hyalinized staghorn-shaped blood vessels [4]. The spindle cells typically have scant cytoplasm, bland nuclei, and occasional nucleoli. Most reported cases of fat-forming SFT shows morphological similarities to classical SFT, except for the presence of varying numbers of mature adipocytes [7, 8]. In our case, the proportion of mature adipose tissue even surpassed that of hemangiopericytomatous vasculature, which posed diagnostic challenges.

Based on the morphology, location, and CT scan findings of the tumor, our case was initially suspected to be an adipocytic neoplasm. However, immunohistochemical staining helped to redirect our diagnosis. We observed strong nuclear and diffuse STAT6 immunoreactivity in tumor cells, along with diffuse positivity for CD34. No immunoreactivity for CK, S100, CD31, ERG, *MDM2*, *CDK4*, Melan A, or HMB45 was detected in the tumor cells. Furthermore, fluorescence in situ hybridization (FISH) did not show amplification of the *MDM2* and *CDK4* genes. As a result, the final diagnosis of the tumor was fat-forming SFT.

The list of soft tissue tumors that should be included in the differential diagnosis includes dedifferentiated liposarcoma (DDLPS), spindle cell lipoma (SCL), atypical lipomatous tumor/well-differentiated liposarcoma (ALT/WDLPS), malignant peripheral nerve sheath tumor (MPNST), and angiomyolipoma (AML). The adipocytic component in fat-forming SFT is mature and lacks the atypical cells seen in dedifferentiated liposarcoma. Additionally, the negative expression of *MDM2* and *CDK4*, as revealed by immunohistochemical staining and FISH analysis, excludes the diagnosis of DDLPS and ALT/WDLPS. Negative immunostaining for HMB45, Melan A, and S100 in tumor cells also excludes angiomyolipoma and malignant peripheral nerve sheath tumor from the differential diagnosis, respectively. Spindle cell lipoma typically exhibits bland spindle cells with uniform and elongated nuclei, mature adipocytes, and ropy collagen bundles. It primarily occurs in the subcutis of the posterior neck, back, and shoulders, and is rarely found in the retroperitoneal area. Expression of CD34 is also observed in spindle cell lipoma, similar to fat-forming SFT. The strong and diffuse STAT6 immunoreactivity is a key diagnostic factor in this case. It is important to note that approximately 11% of DDLPS exhibit nuclear expression of STAT6. This may be due to the close proximity of STAT6 (chr12q13) and MDM2 (chr12q15) on chr12, leading to potential coamplification of STAT6 with MDM2 in DDLPS. This coamplification can result in STAT6 protein expression, which complicates the differential diagnosis [9].

In these situations, the presence of well-differentiated liposarcoma, confirmed *MDM2* and *CDK4* expression by immunohistochemistry, or amplification by FISH would strongly support the diagnosis of dedifferentiated liposarcoma. The sensitivity and specificity of *MDM2* and *CDK4* immunostaining in identifying well-differentiated liposarcoma/dedifferentiated liposarcoma were 97% and 92%, and 83% and 95%, respectively [10]. These immunostainings were particularly useful in differentiating fat-forming SFT from the group of adipose tumors.

Most SFTs exhibit benign histologic features and have a favorable prognosis, but a subset of cases may recur or metastasize. It is crucial to accurately identify patients with the highest risk of recurrence or metastasis during the initial resection to strengthen postoperative monitoring. The development of multivariate risk models has significantly improved the clinical predictive power. Among the recently proposed risk stratification systems, the widely used model for predicting metastatic risk incorporates factors such as mitotic count (≥ 2 mitoses/mm2), patient age (≥ 55 years), and tumor size (stratified into 5 cm tiers) to classify tumors into low, intermediate, and high-risk groups [11, 12]. Tumor location is also an essential prognostic factor, with large tumors within the retroperitoneum being more prone to local recurrence and potentially associated with a poorer outcome compared to tumors in other sites [13]. In our case, the patient has been alive with no evidence of recurrence ten months after tumor resection.

In some cases, fat-forming SFTs may exhibit malignant behavior, which is characterized by the presence of a high-grade component. Rarely, these tumors may also intermix with lipoblasts and/or ALT/WDLPS-like areas, leading to considerable diagnostic confusion with dedifferentiated liposarcoma [14, 15]. The presence of lipoblasts has also been described in some “benign-appearing” fat-forming SFTs [16], but the prognostic significance of such lesions remains controversial. However, lipoblasts and/or ALT/WDLPS-like areas appear to be more frequent in the malignant-appearing subset of fat-forming SFTs, which indicates the need for careful examination to identify any malignant features in cases of fat-forming SFTs [15].

In conclusion, we present a case of fat-forming SFT located in the retroperitoneum. The differential diagnosis with dedifferentiated liposarcoma can be challenging, especially when dealing with retroperitoneal lesions. STAT6 amplification, which occurs in a subset of dedifferentiated liposarcoma, can be a potential pitfall. Therefore, the detection of *CDK4*/*MDM2* by FISH or immunohistochemistry is necessary to exclude dedifferentiated liposarcoma. The diagnosis of fat-forming SFT requires an integrated approach due to its rarity; otherwise, the correct diagnosis could be easily missed, leading to unnecessary treatments for the patient.

## Data Availability

Informed consent was obtained from the patient for research or publications.
